# Increased Growth of a Newly Established Mouse Epithelial Cell Line Transformed with HPV-16 E7 in Diabetic Mice

**DOI:** 10.1371/journal.pone.0164490

**Published:** 2016-10-17

**Authors:** Lan He, Priscilla T. Y. Law, Siaw Shi Boon, Chuqing Zhang, Wendy C. S. Ho, Lawrence Banks, C. K. Wong, Juliana C. N. Chan, Paul K. S. Chan

**Affiliations:** 1 Department of Microbiology, The Chinese University of Hong Kong, Prince of Wales Hospital, Shatin, New Territories, Hong Kong SAR; 2 Department of Medicine and Therapeutics, The Chinese University of Hong Kong, Prince of Wales Hospital, Shatin, New Territories, Hong Kong SAR; 3 Li Ka Shing Institute of Health Sciences, The Chinese University of Hong Kong, Prince of Wales Hospital, Shatin, New Territories, Hong Kong SAR; 4 Department of Chemical Pathology, The Chinese University of Hong Kong, Prince of Wales Hospital, Shatin, New Territories, Hong Kong SAR; 5 Hong Kong Institute of Diabetes and Obesity, The Chinese University of Hong Kong, Prince of Wales Hospital, Shatin, New Territories, Hong Kong SAR; 6 International Centre for Genetic Engineering and Biotechnology, Trieste, Italy; Georgetown University, UNITED STATES

## Abstract

Epidemiological evidence supports that infection with high-risk types of human papillomavirus (HPV) can interact with host and environmental risk factors to contribute to the development of cervical, oropharyngeal, and other anogenital cancers. In this study, we established a mouse epithelial cancer cell line, designated as Chinese University Papillomavirus-1 (CUP-1), from C57BL/KsJ mice through persistent expression of HPV-16 E7 oncogene. After continuous culturing of up to 200 days with over 60 passages, we showed that CUP-1 became an immortalized and transformed epithelial cell line with continuous E7 expression and persistent reduction of retinoblastoma protein (a known target of E7). This model allowed *in-vivo* study of interaction between HPV and co-factors of tumorigenesis in syngeneic mice. Diabetes has been shown to increase HPV pathogenicity in different pathological context. Herein, with this newly-established cell line, we uncovered that diabetes promoted CUP-1 xenograft growth in syngeneic *db*/*db* mice. In sum, we successfully established a HPV-16 E7 transformed mouse epithelial cell line, which allowed subsequent studies of co-factors in multistep HPV carcinogenesis in an immunocompetent host. More importantly, this study is the very first to demonstrate the promoting effect of diabetes on HPV-associated carcinogenesis *in vivo*, implicating the importance of cancer surveillance in diabetic environment.

## Introduction

Persistent infection with high-risk types of human papillomavirus (HPV) is strongly associated with the development of a wide range of malignancies, including cervical, oropharyngeal, anal, vaginal, vulvar and penile cancers. The pathogenic mechanism of HPV has been extensively studied and it is well established that E6 and E7 are its major oncogenes. Interestingly, though infection of high risk HPV types is common, only a small proportion of infected subjects eventually develop cancer [1]. Undoubtedly, interaction of HPV with host risk factors and other environmental factors play an important role in the multistep HPV carcinogenesis. Yet, our knowledge on the correlation between HPV and these co-factors is still very limited.

Diabetes has been shown to increase HPV pathogenicity in different diseases. For instance, patients with type-2 diabetes (T2D) are at increased risk for HPV-associated cancers, including cervical cancer and head and neck cancer [2–4]. In addition, diabetic patients develop more extensive HPV-related genital warts and they are more prone to disease recurrence as compared to non-diabetic subjects [5]. Although epidemiological observations have pinpointed an increased risk of diabetes in HPV pathogenesis, their interaction and underlying molecular mechanisms await further experimental investigation and confirmation. It is therefore pivotal to set up a model to determine if diabetes could act as a co-factor to promote HPV-driven tumorigenesis *in vivo*.

Cell lines represent a valuable tool for studying cellular and molecular responses to a defined condition. They are also essential in establishing xenograft and autograft models to delineate the influence of different host factors (e.g. diabetes) on tumorigenesis in animals. Though ample of HPV-associated human cell lines are available, there is currently very few HPV-related murine epithelial cell lines in market [6]. The most widely used one is TC-1, which was established from mouse lung epithelial cells transformed with HPV-16 E6 and E7 [7]. In this study, we established a baby mouse epithelial cell line, designated as Chinese University Papillomavirus-1 (CUP-1) from C57BL/6 mice through introduction of HPV-16 E7 and determined the effect of diabetes on the growth of CUP-1 xenograft in syngeneic db/db mice.

## Materials and Methods

### Mice

C57BL/KsJ mice, male C57BL/KsJ-+Lepr^db^/+Lepr^db^ diabetic (db/db) mice and non-diabetic control littermates (m+/db) were purchased from Jackson laboratory (Bar Harbor, ME). All animal experimental protocols were reviewed and approved by the Animal Experimentation Ethics Committee of The Chinese University of Hong Kong. The animal study was approved by the Animal Research Ethics Committee of Hong Kong and performed in accordance to the Animals (Control of Experiments) Ordinance (approval No.13/039/MIS). Ketamine/xylazine combinations were administered intraperitoneally for anesthesia. Mice were sacrificed using carbon dioxide overdose. Animals were housed under specific pathogen-free conditions at the Laboratory Animal Service Centre (LASEC) according to the approved protocols and guidelines of the Animal Experimentation Ethics Committee (AEEC) of the Chinese University of Hong Kong.

### CUP-1 cell line establishment

Kidneys of 9-day-old C57BL/KsJ mouse (the same genetic background as db/db mice) were extracted and dispersed into a single-cell suspension by mechanical grinding, followed by digestion with trypsin-EDTA at a concentration of 0.05% in PBS. Primary mouse kidney (BMK) cells were cultured in DMEM, supplemented with 10% fetal bovine serum, 100 U/ml penicillin, 100 U/ml streptomycin and maintained in a humidified incubator at 37°C with 10% CO_2_. Cells were allowed to grow for 48–72 hours to approximately 50% confluency. The HPV-16 E7-expressing construct pJ4Ω-HPV16E7 was transfected into BMK cells by calcium phosphate precipitation. H-ras was co-transfected to augment the immortalization and transforming process as previously described [8–10]. Successfully transfected cells were selected in complete Dulbecco's modified Eagle's medium (DMEM) containing 220 μg/ml G418. Cell colonies presented with epithelial morphology were selected and plated in another vessel for further propagation under phase-contrast microscope to eliminate fibroblast contamination. The culture was maintained in selective medium until it reached 70–80% confluency, which allowed successive passaging. The first cell monolayer grown upon G418 selection was defined as passage 0 on Day 0. After culturing for 200 days with a completion of 60 passages, a stably transformed epithelial cell line with persistent expression of HPV-16 E7, designated as Chinese University Papillomavirus-1 (CUP-1) was obtained.

### Cumulative population doubling determination

Upon subculturing, CUP-1 cells were plated at an initial density of 2 × 10^5^ in a T25 flask and allowed to grow. When cells reached 80% confluency, cell number was determined using Countess Automated Cell Counter (Thermo Fisher Scientific). This process was repeated up to 200 days until the cells reached 60 passages, which is well recognized to represent an infinite lifespan. In each passage, the passage number and date of subculturing were recorded. Cumulative population doubling was determined by the equation: 3.32 x [log(the number of cell harvested)–log(the initial no of cells plated)] + S, where S is the initial population doubling. Cumulative population doubling was then plotted against days in culture to study CUP-1 growth rate. Population doubling time was also calculated according to the equation: log2 (the number of cells harvested/the initial number of cells seeded).

### Quantitative reverse transcription polymerase chain reaction (qRT-PCR)

Cell lysate were harvested for total RNA extraction at every 10 passages from passage 10 (P10) to passage 60 (P60) to ensure continuous E7 expression throughout the whole culturing period. Total RNA was extracted from cell lysate using TRIzol (Thermo Fisher Scientific, Waltham, MA) and treated with DNA-Free^™^ DNase (Thermo Fisher Scientific) to remove genomic DNA carryover. RNA was reverse transcribed to cDNA by poly(dT) primers using Superscript III reverse transcriptase (Thermo Fisher Scientific). HPV-16 E7 levels was determined by qRT-PCR assays using 2x TaqMan universal mix (Thermo Fisher Scientific). The primers used for amplifying HPV-16 E7 were: 5’-ACAAGCAGAACCGGACAGAG-3’ (forward) and 5’-GCCCATYAACAGGTCTTCCA-3’ (reverse). Real-time monitoring of fluorescence was performed on Veriti^™^ 96-Well Thermal Cycler (Thermo Fisher Scientific) with cycling condition conducted at 50°C for 2 minutes, 95°C for 10 minutes, followed by 40 cycles of 95°C for 15 sec and 55°C for 1 minute. Endogenous level of housekeeping β-actin was simultaneously quantified for normalization using 5’-GCACGGATCGTCACCAACT -3’ (forward) and 5’-CATCTTCTCGCGGTGGCCT-3’ (reverse) primers.

### Western blot

Cell lysate were harvested for total protein extraction every 10 passages from P10 –P60. CUP-1 cells were lysed with ice-cold RIPA buffer supplemented with protease inhibitors (Roche Life Science, Germany) and lysates were quantitated using BCA protein assay kit (Thermo Fisher Scientific) according to the manufacturer’s procedures. Twenty microgram of total protein was resolved on a sodium dodecyl sulfate-polyacrylamide gel electrophoresis (SDS-PAGE) and transferred onto polyvinylidene fluoride (PVDF) membrane. The membrane was then blocked with either 5% dry milk or 5% bovine serum albumin (BSA) in Tris buffered saline with Tween20 (TBST) and probed with specific antibodies as follows: E7 (Cervimax, Cat#VS13004), pan-keratin (Cell singaling, Cat#4545), S-100A4 (Abcam, Cat#ab27957) and β-actin (Cell signaling, Cat#12262). Antigen complexes were visualized by ECL chemiluminescence (GE Healthcare, Pittsburgh, PA).

### Immunofluorescence staining

CUP-1 cells plated onto glass coverslips were fixed with methanol and blocked with BSA. Cells were then probed with specific antibodies against pan-keratin (Cell singaling, Cat#4545) and S-100A4 (Abcam, Cat#ab27957), followed by Alexa Fluor-conjugated secondary antibodies (Thermo Fisher Scientific). Finally, cell nuclei were counterstained with 4’,6-diamidino-2-phenylindole (DAPI). Slides were examined using fluorescent microscopy (Leica, Germany) and representative images were captured.

### In vivo xenograft model

Ten million CUP-1 cells were resuspended in 150 μL PBS and injected subcutaneously over the flank of 10-week-old nude mice, diabetic db/db mice and their non-diabetic (m+/db) littermates (10 mice per group). Mice body weight and tumor size were monitored and recorded every other day during the study period. Tumor volume was estimated using the formula (a×b^2^) × 0.5236, where “a” refers to the largest dimension of the tumor and “b” is the perpendicular diameter [11]. Mice were sacrificed when the tumor mass become larger than 2cm^3^. The tumors were paraffin sectioned for hematoxylin and eosin (H&E) staining.

## Results

### CUP-1 cells show steady E7 levels

**Fig 1:**
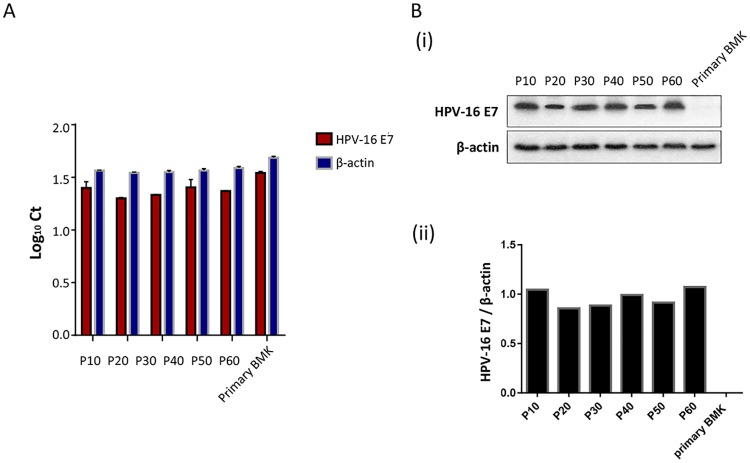
CUP-1 cells stably expressed HPV 16 E7 mRNA and protein. **(A).** HPV16-E7 mRNA levels in CUP-1 cells was confirmed by qRT-PCR analysis at every 10 passages from passage 10 to passage 60 with β-actin as the control. Each column represents the means ± S.D. in three independent experiments. **(B).** HPV16-E7 protein levels in CUP-1 cells was further affirmed by Western blotting. (i) Representative immunoblots showed the expression of 21 kDa HPV16-E7 protein in CUP-1 cells at every 10 passages from P10 to P60 using untransfected BMK cells as a negative control. β-actin was used as a loading control to confirm equal protein loading. (ii) Quantitative analysis of HPV16-E7 protein expression in CUP-1 by ImageJ software. The histogram showed quantified 16E7 levels adjusted with corresponding β-actin level. Each column represents the mean ± S.D from three independent experiments.

Primary cells have a finite life span. They will eventually undergo senescence and apoptosis upon culturing. In this study, a continuous mouse epithelial cell line, designated as CUP-1 was formed by introducing HPV-16 E7 oncogene into primary baby mouse kidney cells. Steady E7 mRNA levels in CUP-1 throughout early passages (Passage 10) to late passages (Passage 60) were confirmed by qRT-PCR (Fig 1A). Continuous E7 protein expression throughout the entire culturing period was further affirmed by Western blotting (Fig 1B and 1C), indicating that CUP-1 cells stably expressed HPV 16 E7.

### CUP-1 cells is immortalized and gained anchorage independent growth

**Fig 2:**
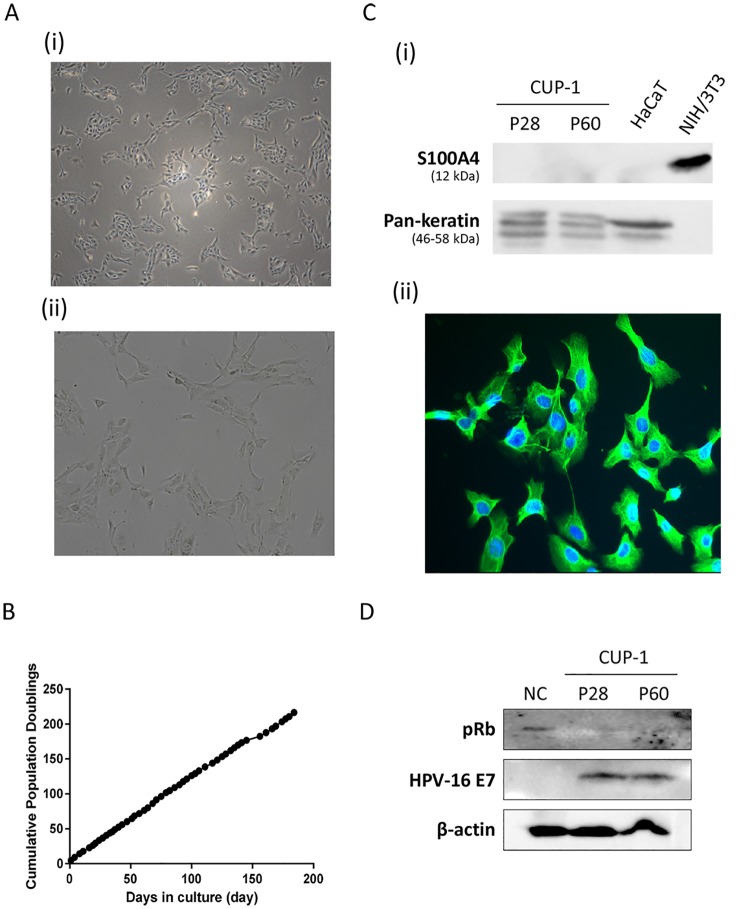
CUP-1 was an immortalized epithelial cell line with functional E7 protein. **(A)** Phase-contrast micrograph of CUP-1 cells, (i) x40 (ii) x200. **(B)** Proliferation profile of CUP-1. CUP-1 was plated at a density of 2 × 10^5^ cells in a T25 flask. When they reached 80% confluency, cell number was determined and cells were subcultured. Each sub-culture was counted as one passage. This process was repeated up to 200 days with over 60 passages. Cumulative population doublings was calculated using the equation: 3.32 x [log(the number of cell harvested)–log(the initial number of cells plated) + S, where S is the initial population doubling. Cumulative population doubling was then plotted against days in culture. Each point represents the mean of triplicate determinations. CUP-1 grew in a steady rate without any sign of senescence, indicating that it was immortalized. **(C)** Cell type of CUP-1 at passage 28 (P28) and 60 (P60) was characterized via (i) Western blotting and (ii) immunofluorescence using keratinocyte marker pan-keratin (green) and fibroblast marker S100A. HaCaT (keratinocytes) and NIH 3T3 (fibroblasts) were included as cell type controls. CUP-1 nuclei was counterstained by DAPI (blue) in immunofluorescence. **(D)** Western blot analysis of pRb levels in CUP-1. Total cell lysate was collected from non-transfected primary baby mouse kidney (BMK) cells and CUP-1 cells at P28 and P60. CUP-1 expressed E7 and readily degraded pRb in both P28 and P60. β-actin was included as the loading control.

Proliferation profile of CUP-1 revealed that it did not show any sign of crisis upon transfection and grew continuously with a steady rate over the whole study period. CUP-1 cells did not show any sign of senescence up to 200 days of culture with over 60 passages (Fig 2B), indicating that they achieved infinite life span and were indeed immortalized. Doubling time of CUP-1 was approximately 18 hours at passage 60. On the other hand, control cells transfected with empty plasmid were unable to survive after passage and died within 20 days of culture.

Morphologically, CUP-1 cells displayed a spindle and dendritic-like shape in tissue culture vessel (Fig 2A). They developed increasing number of dendritic-like structures protruding from the cells throughout the culture period. Interestingly, unlike primary cells which grow in monolayer, CUP-1 cells were able to pile up on top of each other and were able to form foci upon reaching full confluency (data not shown), implicating that they lost cell-cell contact inhibition.

To avoid fibroblast contamination, only colonies presented with epithelial morphology were selected for subculture under phase contrast microscopy. The cell type of CUP-1 was further affirmed by Western blotting and immunofluorescence using pan-keratin as a specific epithelial marker and S100A4 as a fibroblast marker. Results showed that CUP-1 cells expressed high levels of pan-keratin in cytoplasm (Fig 2C and 2D), whilst S100A4 was not detectable (Fig 2C), indicating that CUP-1 was of epithelial origin.

The most well-characterized E7 properties which contributes to its oncogenic action is its ability to degrade pRb and related pocket proteins. Consistent with E7 expression, Western blotting showed that pRB was degraded in CUP-1 cells both in P28 and P60 (Fig 2E), indicating E7 exerted a functional role in CUP-1 in abrogating pRb related functions and resemble E7 functions as if in human cells.

### CUP-1 cells induced tumor formation in nude mice

**Fig 3:**
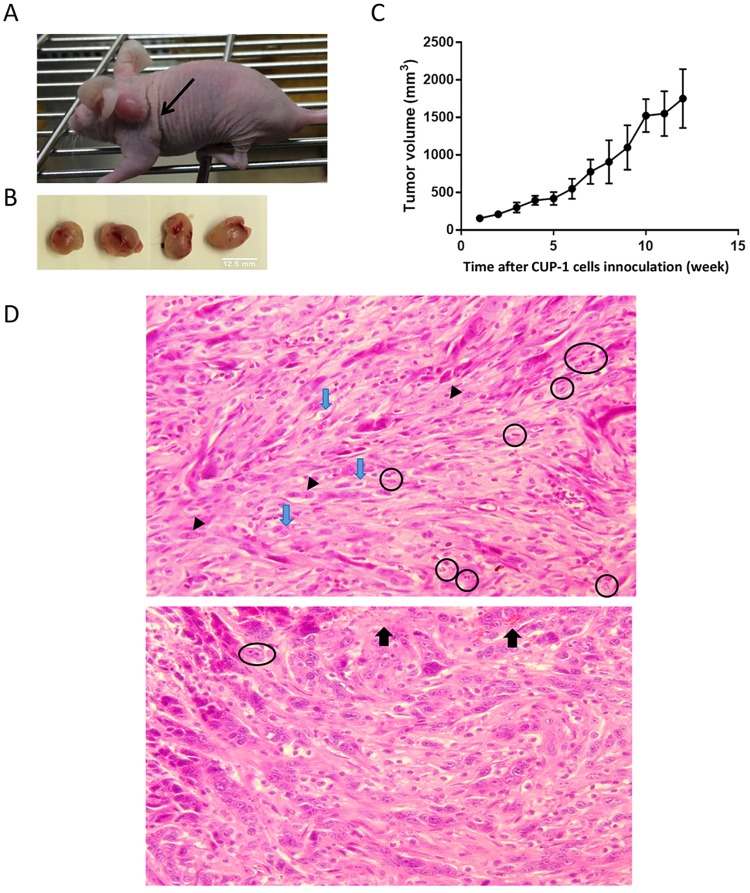
CUP-1 cells were tumorigenic in athymic (nu/nu) mice. Ten million CUP-1 cells were injected subcutaneously into the scruff of 10-week-old nude mice and tumour development was monitored for 12 weeks. **(A)** Representative image of a nude mouse with significant tumor mass growth (arrow) at week 12 upon CUP-1 inoculation. **(B)** Representative excised tumors at 12 weeks after inoculation. **(C)** CUP-1 xenograft growth in nude mice. Tumor volumes were monitored every other day over the study period by caliper measurement of the largest dimension of the tumor, “a” and the perpendicular diameter, “b”. Tumor volumes were calculated using the formula (a×b^2^) × 0.5236. Each point represents mean ± S.E.M (n = 10). **(D)** (i) and (ii) Representative photomicrographs of H&E-stained CUP-1 tumor tissues excised from nude mice, X200. Tissue sections showed presence of cells with enlarged and pleomorphic nucleus (black arrowhead), mitotic figures (black circle) and infiltration of red blood cells (black arrow).

Upon subcutaneous injection of CUP-1 cells over the flank of nude mice, a tumor mass was formed (Fig 3) and it continued to grow with persistent increase in volume over the study period (Fig 3B). At week 12, the tumors in some of the mice became oversized (larger than 2cm^3^). Mice were therefore sacrificed by carbon dioxide asphyxiation and tumors were excised. The dissected tumors appeared to be irregular in shape and had blood vessels on the surface (Fig 3C).

Subsequent histological examination indicated that the excised tumor showed presence of pleomorphic and enlarged nucleus, increased nucleoli, increased nuclear-cytoplasmic ratio and mitotic figures. Infiltration of red blood cells was also observed, indicating possible angiogenesis (Fig 3D).

### Diabetic (db/db) mice promoted CUP-1 xenograft growth

**Fig 4:**
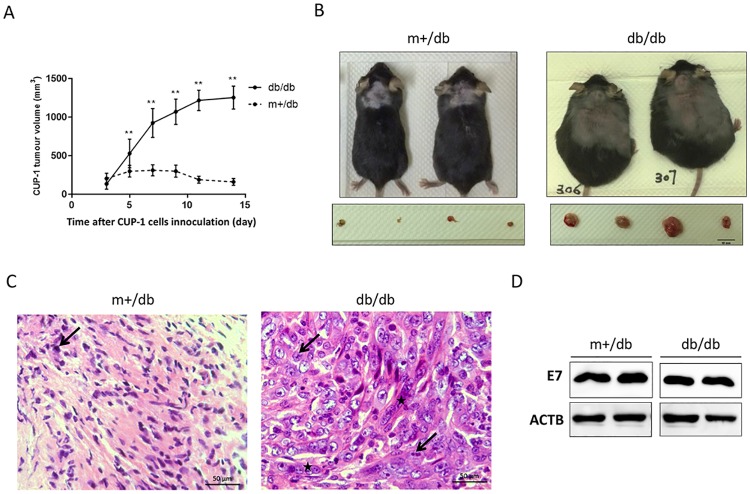
Diabetic (db/db) mice promoted CUP-1 xenograft growth. CUP-1 cells were injected subcutaneously into the scruff of diabetic db/db mice and non-diabetic control littermates (m+/db) of 10 weeks age. Tumor size was monitored every other day for 2 weeks and tumor volume was calculated as in nude mice. **(A)** CUP-1 tumor growth in db/db and m+/db mice. Each point represents mean ± S.E.M (n = 10). **(B**) Representative photographs show db/db (right) and m+/db mice (left) with respective representative excised tumors on day 27 after CUP-1 inoculation. Arrows indicate the site with CUP-1 inoculation. **(C)** Representative photomicrographs of H&E-stained CUP-1 tumor tissues from db/db (left) and m+/db mice (right) excised on day 27 after CUP-1 inoculation, X400. Atypical cells with large irregular nuclei (stars) and mitotic figures (arrows) were present in CUP-1 tumor tissues. **(D)** Western blotting confirmed E7 expression in CUP-1 excised xenograft from both db/db and m+/db mice.

To study the effects of diabetes on HPV-16 E7-mediated tumorigenesis, CUP-1 cells were injected subcutaneously over the scruff of diabetic db/db mice and non-diabetic m+/db littermates. Similar with nude mice, visible tumor mass appeared on day 3 upon subcutaneous inoculation of CUP-1 cells. Interestingly, growth kinetics of CUP-1 xenograft differed significantly between the two groups. From day 3 to 7 after CUP-1 cell inoculation, there was a 6-fold increase in tumor volume in db/db mice. In contrast, tumor volume increased only 1.5-fold in non-diabetic m+/db mice from day 3 to 7 and started to regress on day 7 (Fig 4A). CUP-1 tumors were completely cleared in all m+/db mice on day 29. The tumor volume of db/db mice was nearly 4-fold larger than that of m+/db mice on day 14 (1256.9±31.4 vs 314.4±22.1 mm^3^, P<0.001).

Further histological examination of excised tumours (Fig 4C) showed that CUP-1 xenograft from both db/db and m+/db mice exhibited an increased nuclear-cytoplasmic ratio and cell density, which are common features associated with dysplasia and malignant cells. Nuclear pleomorphism and mitotic figures were markedly increased in tumor tissues excised from db/db mice, implicating increased mitosis and excessive proliferation featuring malignant neoplasms. Further western blotting of excised CUP-1 xenograft confirmed successful E7 expression in both db/db and m+/db mice. Collectively, db/db mice promoted CUP-1 xenograft growth and delayed tumor clearance.

## Discussion

In this study, we successfully established a murine epithelial cell line, designated as CUP-1 by introduction of HPV-16 E7. CUP-1 have been continuously cultured for up to 200 days with over 60 passages, indicating that it gained infinite lifespan. Stable and continuous E7 expression from early to late passages was confirmed by qRT-PCR and Western blot. Unlike human keratinocytes transformed with HPV-16 E6/E7 [12], CUP-1 cells did not show any sign of crisis and grew continuously with a steady rate over the whole study period.

After reaching full confluency, CUP-1 cells lost cell-cell contact inhibition and were able to form foci. This is in agreement with previous reports, in which the same phenomenon was also observed and is likely to be contributed by E7 expression in association with p600 to resist anoikis [13–15]. Functional role of E7 in CUP-1 was further affirmed by Western blotting. pRb degradation is the most well-characterized E7 properties that contributes to its oncogenic action. It has been well-proved that the binding of E7 to pRb is mediated by a conserved Leu-X-Cys-X-Glu (LXCXE) motif in the conserved region 2 (CR2) of E7 and this domain is both necessary and sufficient for E7/pRb association [16]. Consistently, Western blotting showed that pRB was persistently degraded in CUP-1 cells. Upon pRb degradation, E2F family of transcription factors is released and will in turn lead to subsequent activation of genes promoting cell proliferation. The tumorigenicity of CUP-1 was demonstrated by the successful establishment of xenograft in nude mice. Collectively, these results showed that CUP-1 displayed carcinogenic characteristics and represented one of the very few HPV immortalized and transformed murine epithelial cell lines, with continuous HPV-16 E7 expression and functional E7 activity.

Though the pathogenic mechanism of HPV has been extensively studied, questions remain over its interaction with environmental and host risk factors in tumorigenesis. Several studies have suggested that type 2 diabetes is associated with increased risk of cervical cancer and head and neck cancer which are related to HPV infection [2]. Therefore, diabetic microenvironment might be an important co-factor that promotes HPV-driven tumorigenesis. Navarro-Meza *et al* [3] reported that hyperglycemia facilitated HPV infection and might be a risk factor for development of low grade cervical lesions. In support of this notion, increased incidence of cervical cancer had been reported in women with type 2 diabetes from Spain, Japan [17] and Mexico [18].

Using CUP-1, we successfully delineated the promoting effects of diabetes to HPV-16 E7-mediated tumorigenesis in db/db mice. Our results implicated the importance of cancer surveillance in this patient subgroup. More importantly, this implies the possibility of anti-diabetic drug as a therapeutic option for HPV-related cancers in diabetes patients. The underlying mechanism of this observation, however, awaits further investigation.

Similarly, CUP-1 could be used to study the effects of other host or environment co-factors to HPV-mediated tumorigenesis in any host with the same C57 genetic background. For instances, several epidemiologic studies have pinpointed the effects of environmental pollutants generated by cigarette smoking [19], heavy traffic [20] and even cooking oil fumes [4] on cervical cancer development. The establishment of CUP-1 provides a simple way for *in vivo* examination of the involvements of these co-factors on HPV, in particular E7, -mediated tumorigenesis. The advantage of using CUP-1 xenograft model instead of traditional nude mice xenograft model is that it allows *in vivo* studies in an immunocompetent host and can take into account the effect of the host immune system.

Our present study successfully established a murine epithelial cell line, CUP-1 transformed with HPV-16 E7 and with functional E7 properties. With CUP-1, we successfully uncovered the promoting effects of diabetes in HPV-mediated tumorigenesis. This cell line represents an important tool for studying the effects of co-factors in HPV-mediated carcinogenesis, and also provides a translational platform for preclinical screening of novel therapeutics.
